# Results of the phase I CCTG IND.231 trial of CX-5461 in patients with advanced solid tumors enriched for DNA-repair deficiencies

**DOI:** 10.1038/s41467-022-31199-2

**Published:** 2022-06-24

**Authors:** John Hilton, Karen Gelmon, Philippe L. Bedard, Dongsheng Tu, Hong Xu, Anna V. Tinker, Rachel Goodwin, Scott A. Laurie, Derek Jonker, Aaron R. Hansen, Zachary W. Veitch, Daniel J. Renouf, Linda Hagerman, Hongbo Lui, Bingshu Chen, Deb Kellar, Irene Li, Sung-Eun Lee, Takako Kono, Brian Y. C. Cheng, Damian Yap, Daniel Lai, Sean Beatty, John Soong, Kathleen I. Pritchard, Isabel Soria-Bretones, Eric Chen, Harriet Feilotter, Moira Rushton, Lesley Seymour, Samuel Aparicio, David W. Cescon

**Affiliations:** 1 Ottawa Hospital Research Institute, Ottawa, ON Canada; 2 BC Cancer - Vancouver Centre, Vancouver, BC V5Z 1L3 Canada; 3 Princess Margaret Cancer Centre, University Health Network, Toronto, ON Canada; 4 Division of Medical Oncology, Department of Medicine, University of Toronto, Toronto, ON Canada; 5 Canadian Cancer Trials Group, 10 Stuart Street, Kingston, ON K7L 3N6 Canada; 6 Molecular Oncology, BC Cancer Research Institute, Vancouver, BC V5Z 1L3 Canada; 7 Senhwa Biosciences, Inc, Taipei, Taiwan; 8 Sunnybrook Health Sciences Centre, Toronto, ON Canada; 9 Pathology and Laboratory Medicine, UBC, Vancouver, BC Canada

**Keywords:** Phase I trials, Targeted therapies, Cancer therapy

## Abstract

CX-5461 is a G-quadruplex stabilizer that exhibits synthetic lethality in homologous recombination-deficient models. In this multicentre phase I trial in patients with solid tumors, 40 patients are treated across 10 dose levels (50–650 mg/m^2^) to determine the recommended phase II dose (primary outcome), and evaluate safety, tolerability, pharmacokinetics (secondary outcomes). Defective homologous recombination is explored as a predictive biomarker of response. CX-5461 is generally well tolerated, with a recommended phase II dose of 475 mg/m^2^ days 1, 8 and 15 every 4 weeks, and dose limiting phototoxicity. Responses are observed in 14% of patients, primarily in patients with defective homologous recombination. Reversion mutations in PALB2 and BRCA2 are detected on progression following initial response in germline carriers, confirming the underlying synthetic lethal mechanism. In vitro characterization of UV sensitization shows this toxicity is related to the CX-5461 chemotype, independent of G-quadruplex synthetic lethality. These results establish clinical proof-of-concept for this G-quadruplex stabilizer. Clinicaltrials.gov NCT02719977.

## Abstract (web summary)

G-quadruplex stabilizers, including CX-5461, exhibit synthetic lethality with loss of BRCA1/2 in preclinical models. Here the authors report the results of a phase I study of CX-5461 in patients with solid tumors enriched for DNA-repair deficiencies.

## Introduction

G-quadruplexes (G4) are tertiary DNA structures, which are believed to form at over 700,000 guanine-rich regions of the genome^1–4^. Especially prevalent in telomeres, promoters, and 5’-untranslated regions, neighboring guanine tetramers can stack and form higher-order DNA complexes. The exact function of G-quadruplexes in the human genome is unknown, but these may function in telomere maintenance, gene regulation, and chromatid pairing in meiosis^1^. In normal cells, G4 structures are likely transient and easily resolved by helicases^5^; however, when not properly resolved, G4 structures can cause replication fork arrest or DNA breaks. These events require homologous recombination repair (HR-repair) to resolve^5,6^. It has been shown that G4 ligand tool compounds such as pyridostatin (PDS) are synthetic lethal with loss of HR repair, suggesting a biomarker-driven therapeutic hypothesis^7^.

Loss of HR repair, which is essential for error-free repair of double-stranded DNA breaks, is a common finding in both hereditary and sporadic malignancies, including a subset of breast, ovarian, pancreatic, and prostate cancers^8^ and is a therapeutic vulnerability. Most commonly, HR-repair deficiency (HRD) involves homozygous inactivation of the *BRCA1* and *BRCA2* genes, but this phenotype can also be observed with loss of function of other proteins such as PALB2, ATM (in certain contexts), and MRE11^9^. Currently, Poly (ADP-ribose) polymerases (PARP) inhibitors and platinum-based chemotherapy are used in this setting, as HRD confers sensitivity to these agents by a synthetic lethal mechanism^10,11^. Overlapping hematologic toxicities seen with such agents limits the development of therapeutic combinations, and inevitable resistance to these classes of drugs occurs, motivating the development of new treatment options for this group of patients.

We have recently shown that CX-5461, a fluoroquinolone ellipticine derivative of CX-3543^12^, originally identified as an RNA-Pol1 inhibitor with potential in hematologic cancers, is a potent G4 binder and stabilizer in vitro and in vivo independently of its RNA-Pol1 inhibitory activity. CX-5461 stabilizes G4 in DNA, resulting in replication fork collapse and single-stranded breaks, which convert to double-stranded DNA breaks. CX-5461 was found to be synthetically lethal in *BRCA2* and *BRCA1*-deficient tumor models both in vitro and in vivo, independently of RNA polymerase 1 inhibition^3^. G4 stabilization with CX-5461 could thus represent a novel therapeutic strategy for cancers with germline or somatic defects in HR-repair^7^.

In a prior study of CX-5461 in patients with hematologic malignancies, 170 mg/m^2^ intravenously once every 3 weeks (q3w) was recommended for further study, based on dose-limiting toxicities palmar–plantar erythrodysesthesia and phototoxicity^13^. Preclinical modeling and clinical pharmacokinetics suggested that more frequent administration could be desirable in solid tumors.

To advance clinical evaluation of G4 stabilization in HRD patient populations, we conducted a phase I biomarker-driven clinical trial of CX-5461 in patients with solid tumors, exploring alternative dosing schedules and preferentially enrolling patients with HRD.

Here we show that CX-5461 is generally well-tolerated, with a recommended phase II dose of 475 mg/m^2^ days 1, 8, and 15 every 4 weeks, and dose-limiting phototoxicity. Antitumor activity is observed primarily in patients with HR-defective tumors, and reversion mutations in *PALB2* and *BRCA2* are observed in association with the development of acquired resistance. Notably, the dose-limiting photosensitivity observed with CX-5461 arises from a mechanism independent of its G4-stabilizing effect. Altogether, we establish clinical proof-of-concept for this synthetic lethal strategy for HR-deficient solid tumors.

## Results

Forty-one patients were enrolled between 13 June 2016 and 26 August 2019, one of whom was withdrawn prior to receiving treatment (DL9). All treated patients were evaluable for toxicity and response (1 patient had non-measurable disease only but was considered evaluable for non-CR/non-PD and PD); 4 were not reassessed after baseline and were thus inevaluable for response (two patients discontinued therapy (symptomatic progression, patient refusal) but died prior to reassessment, two patients discontinued CX-5461 (symptomatic progression, toxicity) and started other therapies prior to reassessment). The most common tumor type was breast (*n* = 19), followed by ovarian (*n* = 7) and pancreatic (*n* = 3) cancers, and 78% of participants were female. Based on clinical germline or tumor testing performed prior to enrollment, 19 patients had a germline BRCA1 (*n* = 6) or BRCA2 (*n* = 13) mutation, and 1 had a germline PALB2 mutation; 2 patients had a BRCA2 variant of uncertain significance (VUS) and 1 patient had a PALB2 VUS. Three others had somatic BRCA1/2 (*n* = 3) mutations. The median number of prior lines of therapy was 4. A summary of patient characteristics is presented in Table 1.

**Table 1:** Patient characteristics.

		Number	%
Median age (range)		53 (25–73)	
Sex	Female	31	77.5
	Male	9	22.5
Performance status (ECOG)	0	8	20
	1	30	75
	2	2	5
Malignancy type	Breast	19	47.5
	Ovary	7	17.5
	Pancreas	3	7.5
	Lung cancer: non-small cell	2	5
	Other*	9	22.5
Prior therapy	Chemotherapy	40	100
	Hormone therapy	14	35
	Immunotherapy	8	20
	Radiotherapy	24	60
	PARP inhibitor**	8	20
	Prior platinum	32	80
	Other systemic therapy	11	27.5
	Other therapy	5	12.5
Number of prior lines of therapy	1	4	10
	2	4	10
	3	11	27.5
	≥4	21	52.5
Number of prior chemotherapy regimens–neoadjuvant	1	7	17.5
	2	1	2.5
Number of prior chemotherapy regimens–adjuvant	1	14	35
	2	1	2.5
Number of prior chemotherapy regimens–advanced/metastatic disease	1	9	22.5
	2	11	27.5
	3	9	22.5
	≥4	8	20
Mutation Status (known at study enrollment)	Germline BRCA1	6	15
	Germline BRCA2***	13	33
	Somatic BRCA1 or 2	3	8
	PALB2 ***	1	2.5

Forty patients were enrolled and treated in the study.

*Other tumor type includes one of each: adrenal, anal, appendix, biliary, colon, head and neck, small intestine, soft tissue sarcoma, and uterine sarcoma.

**Includes one patient treated on the blinded trial of PARP inhibitor (niraparib vs placebo) and not unblinded.

***Two additional pts had BRCA2 VUS and 1 pt PALB2VUS.

### Dose escalation and adverse events

Ten dose levels (DL0–9) and 2 schedules were explored in this study (Table 2). The median number of cycles administered was two (range: 1–16). The most common adverse events (AE) considered related to CX-5461 were skin phototoxicity and nausea. The only grade 3 or 4 related AEs were skin phototoxicity (15%), palmar–plantar erythrodysesthesia syndrome (2.5%), and nausea (2.5%). Grade 1 or 2 mucositis, palmar–plantar erythrodysesthesia syndrome, eye phototoxicity, headache, and dry eyes were also reported (Tables 3–5). Skin phototoxicity was observed at all dose levels but ocular phototoxicity appeared to be dose-related, occurring in one patient at DL1 and then at DL6 and above.

**Table 2:** Dose levels, schedule, and dose-limiting toxicity.

Dose level	CX-5461 dose (mg/m^2^/dose)	Schedule (Q4 weeks)	Planned dose/week (mg/m^2^)	Number enrolled	Dose-limiting toxicities
0	50	Day 1 & 8	25	4	
1	100		50	4	
2	150		75	4	
3	200		100	4	
4	250		125	3	
5	325		163	3	
6	475		238	3	
7	325	Day 1, 8 & 15	244	4	
8	475*		356	6	
9	650		488	5	2 **

*Includes 1 patient treated in the expansion phase.

**One occurred after cycle 1 (phototoxicity (skin and eye)).

**Table 3:** Select non-hematologic adverse events of any causality by dose level: all grades.

Dose (mg/m^2^)	50	100	150	200	250	325	475	325	475*	650
Schedule	d1,8	d1,8	d1,8	d1,8	d1,8	d1,8	d1,8	d1,8,15	d1,8,15	d1,8,15
*N patients*	4	4	4	4	3	3	3	4	6	5
Dry eye	1	1	2	1				1		1
Eye phototoxicity		1					1	1	2	3
Diarrhea	2	1	1	2		1			1	1
Mucositis oral		2	1		1		1			2
Nausea	3	3	4	3	2	2 (1)	1	1	6	3
Fatigue	4	3	3	2	2	3 (1)	2	2	5 (2)	3 (1)
Headache	1	3	2		1				2	1
Palmar–plantar erythrodysesthesia syndrome	1	1	1		2	1	1	1	1	1 (1)
Phototoxicity	2 (1)	1	1	2	3 (1)	1	3	4 (1)	4 (2)	3 (1)

Forty patients were enrolled and treated with CX-5461.

*Includes one patient treated in the expansion phase.

**Table 4:** Hematology and biochemistry.

Dose (mg/m^2^)	50	100	150	200	250	325	475	325	475*	650
Schedule	d1,8	d1,8	d1,8	d1,8	d1,8	d1,8	d1,8	d1,8,15	d1,8,15	d1,8,15
*N pts*	4	4	4	4	3	3	3	4	6	5
Alkaline phosphatase	–	–	1	–	–	–	–	–	–	1
Bilirubin	–	–	1	–	–	–	–	–	–	–
AST	–	–	2	1	–	–	–	1	–	–
ALT	–	–	2	1	–	–	–	–	–	–
Anemia	–	–	–	1	–	–	–	–	2	–
Neutropenia	–	–	1	–	–	–	–	1	–	2
Thrombocytopenia	–	–	1	–	–	–	–	–	–	–

Grade 3 or 4 by dose level. Forty patients were enrolled and treated with CX-5461.

*Includes 1 patient treated in the expansion phase.

**Table 5:** Treatment-related non-hematologic adverse events were reported in ≥10% of patients treated with CX-5461 in the study*.

Description	Grade 1–2		Grade 3–4		Total	
	N	%	N	%	N	%
Dry eye	4	1%	0	0%	4	10%
Eye phototoxicity	8	20%	0	0 %	8	20%
Headache	5	12.5%	0	0%	5	12.5%
Mucositis oral	6	15%	0	0%	6	15%
Nausea	17	42.5%	1	2.5%	18	45%
Palmar–plantar erythrodysesthesia	8	20%	1	2.5%	9	22.5%
Phototoxicity (skin)	18	4%	6	15%	24	60%

Forty patients were enrolled and treated with CX-5461.

^*^There were no treatment-related grade 5 AEs.

Sixteen serious AEs (SAEs) were reported in 13 patients, 3 were considered to be related to CX-5461 (grade 3 phototoxicity (DL0), grade 2 phototoxicity of the eye (DL8), grade 3 phototoxicity (DL8)). None of these 3 SAEs were considered DLT, as protocol-specified maximal UV protection was not used. At DL9 (650 mg/m^2^ d1, 8, 15), 1 patient had grade 3 skin phototoxicity; one patient had grade 2 eye phototoxicity in cycle 2 and grade 2 skin phototoxicity and grade 3 palmar–plantar erythrodysesthesia during cycle 3; one patient had grade 2 eye phototoxicity and grade 3 skin phototoxicity during cycle 1. After a review of the data and discussion among investigators, this dose level was considered the maximal administered dose (MAD), and DL8 (475 mg/m^2^ d1, 8, 15) was declared the recommended phase 2 dose (RP2D).

### Pharmacokinetics

**Fig. 1:**
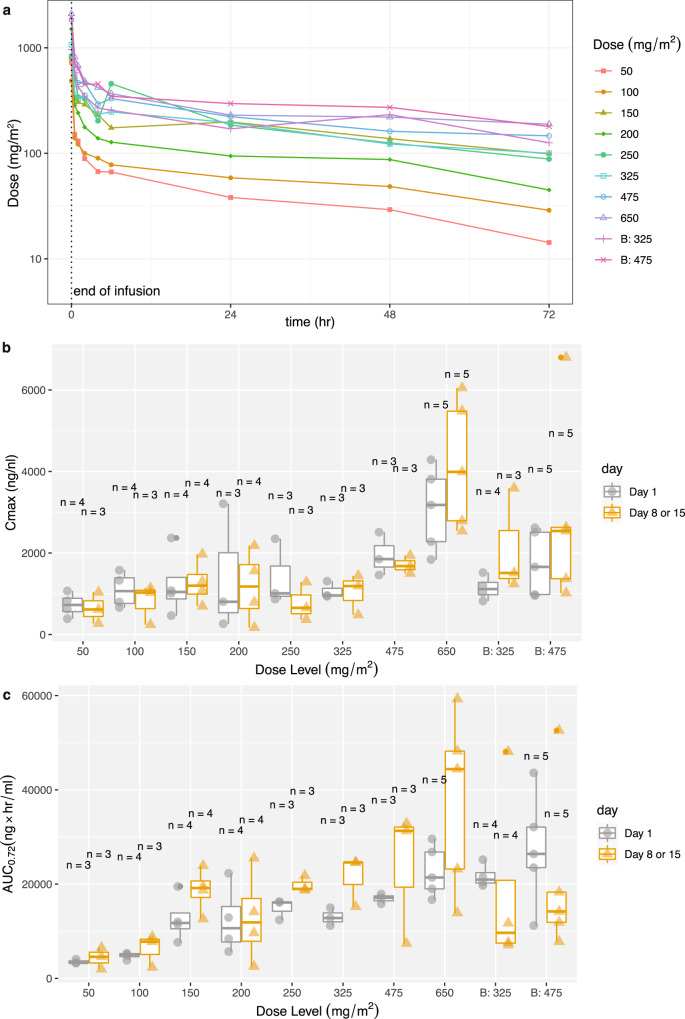
Plasma pharmacokinetics of CX-5461 administration. **a** Day 1 CX-5461 concentration time-course from serial sampling for each dose level: 50 mg/m^2^ (*n* = 3), 100 mg/m^2^ (*n* = 4), 150 mg/m^2^ (*n* = 4), 200 mg/m^2^ (*n* = 4), 250 mg/m^2^ (*n* = 3), 325 mg/m^2^ (*n* = 3), 475 mg/m^2^ (*n* = 3), 650 mg/m^2^ (*n* = 6), B:325 mg/m^2^ (*n* = 6), B:475 mg/m^2^ (*n* = 4). **b**, **c** *C*_max_ (mean, SD), and AUC (mean, SD) for each dose level, sampled on the days shown. PK was performed on cycle 1 day 1 for all patients. Repeat samples were performed on cycle 1 day 8 for patients on a days 1, 8 schedule and on day 15 for those on a days 1, 8, 15 schedule. Repeat sample size indicated as *n* = above each group. Source data are provided as a Source Data file.

The mean CX-5461 concentration at each dose level is shown in Fig. 1a. CX-5461 exposure, as measured by maximum concentration (*C*_max_) and AUC_0-72_, increased with each DL (Fig. 1). However, there was large inter- and intra-subject variability, which, together with the small number of patients treated at each DL, makes dose proportionality difficult to assess. CX-5461 was eliminated slowly, supporting once-weekly dosing. At the RP2D, the average day 1 *C*_max_ was 1745 ± 801 ng/mL, and day 15 *C*_max_ was 2872 ± 2307 ng/ml with an elimination half-life (*t*_1/2_) of 61.5 ± 15.5 h and 59 ± 11.2 h, respectively. The mean trough plasma level taken prior to cycle 2 day 1 (14 days after the prior dose) was 49.1 ± 34.5 ng/ml, indicating minimal residual CX-5461.

### Patient and tumor genotypes

**Fig. 2:**
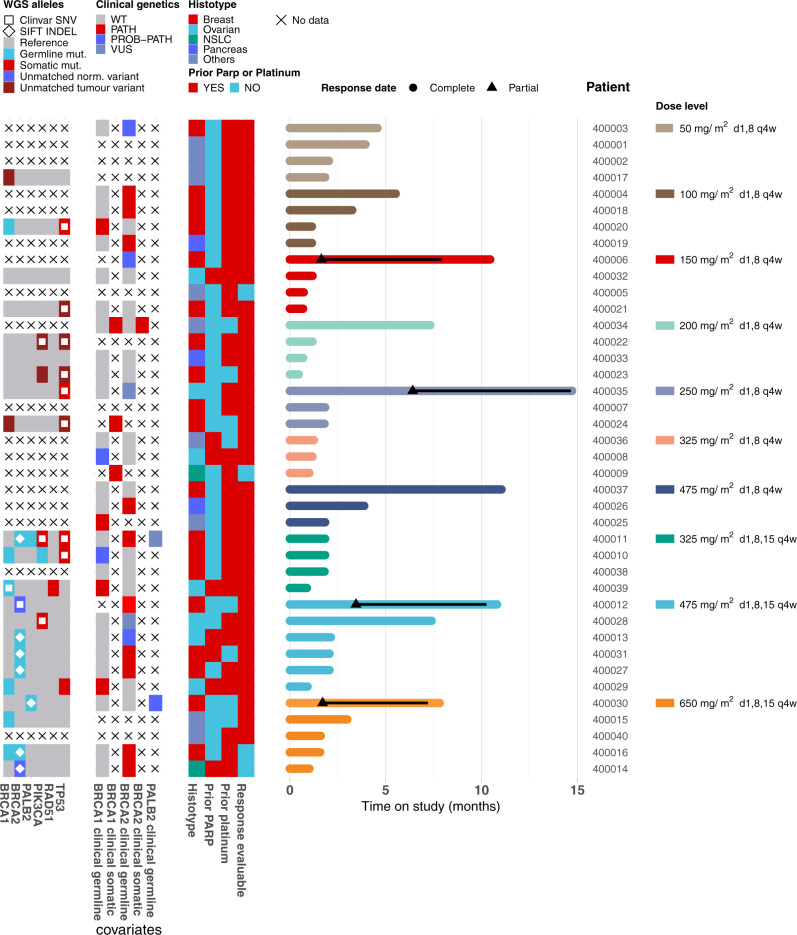
Combined waffle-swimmers plot for genotypes, clinical characteristics, and duration of responses for patients treated with CX-5461. Left waffle panel WGS functional classification; color box; cosmic/fathmm SNV or indel; color box with diamond, SIFT-indel pathogenic; color box with square clinvar pathogenic SNV. Left-center panel clinical germline testing alleles reported as pathogenic/prob-pathogenic, VUS, WT for *BRCA1/2* and *PALB2*. Center-right waffle panel, histotypes, prior-treatment, response evaluable; Right panel, swimmers plot of responses by dose level. Solid colour lines, time on the study (months); solid black line inset, duration of response, response end at end of the black line. *n* = 40 patients, source data are provided as a Source Data file.

Clinical testing reports for germline pathogenic aberrations for *BRCA1*, *BRCA2*, *PALB2* were available for 32 patients. These results were primarily from hereditary cancer screening programs, and thus patients may have had other aberrations not detailed in the clinical report. Alleles classed by clinical testing as pathogenic, likely pathogenic, or VUS, were coded for *BRCA1*, *BRCA2*, *PALB2* (Fig. 2, Supplementary Table 1). Cases/loci without allele information were coded as not applicable (NA)/missing data. For the 20 patients with available formalin-fixed paraffin-embedded (FFPE) tumor material, whole-genome sequencing (WGS) was performed to capture any additional pathogenic/likely pathogenic alleles, as described in WGS methods (Fig. 2, Supplementary Table 1). For 14 cases with matched tumor-normal, germline and somatic protein-coding mutations (SNV, indels) for *BRCA1*, *BRCA2*, *PALB2,* and *TP53*, were coded as pathogenic/likely pathogenic (Cosmic SNV/Indel pathogenic, ClinVar SNVs, SIFT-indel) and for six tumors without matched normal, pathogenic/likely pathogenic mutations in *BRCA1*, *BRCA2*, *PALB2*, *TP53* were coded as present, germline-somatic status unknown (Fig. 2). WGS of normal tissue was performed in two additional patients, without matched tumors. Pathogenic/likely pathogenic mutations from WGS in other loci were sporadic/low frequency. Both matched and unmatched samples sent for WGS identified to have only reference alleles at the genes of interest were coded as “Reference”.

### Efficacy

**Fig. 3:**
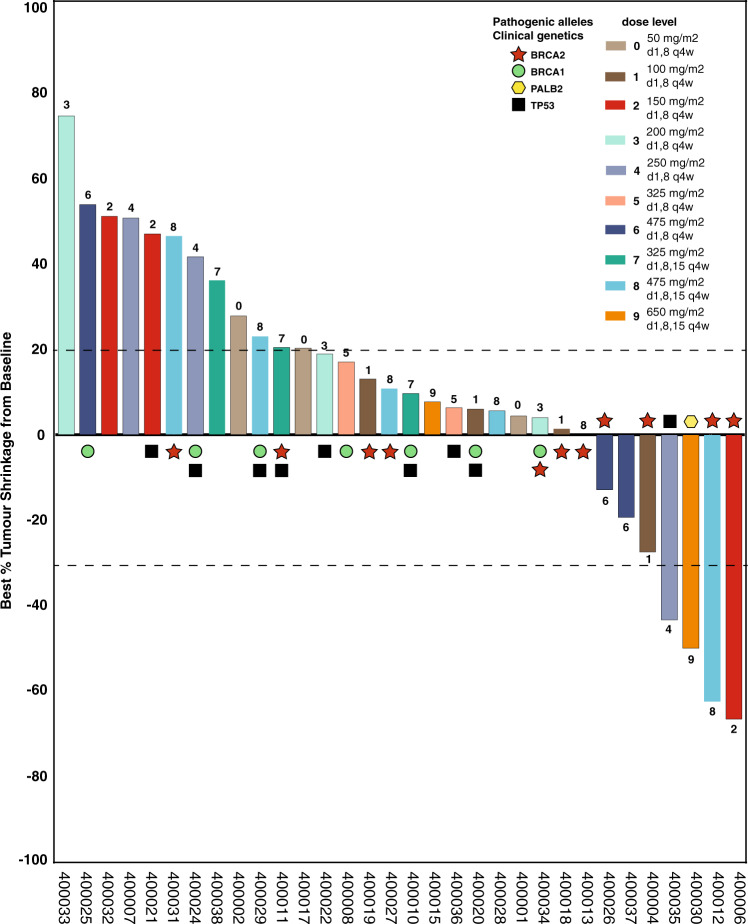
Best response in target lesions for evaluable patients. Objective response (all PR) was observed in 4 of 32 evaluable patients treated with CX-5461. Color bars indicate dose levels per key, numeric dose level above/below bars. Symbols denote germline and/or tumor somatic genotypes (star *BRCA2*, circle *BRCA1*, hexagon *PALB2*, square *TP53* (from WGS only)). Source data are provided as a Source Data file.

Four patients had confirmed PR (3 patients with breast and 1 with ovarian cancer) (ORR 4/40 = 10%, 95% CI: 2.8–23.7); all patients with PR had germline DNA-repair abnormalities (2 *BRCA2*, 1 *PALB2*, 1 *TP53* (with a concurrent *BRCA2* VUS) (Figs. 2, 3). Among patients with tumor types where germline alterations in HR genes (including *BRCA1*/*BRCA2*/*PALB2*) have been shown to have a functional role (breast, ovarian, pancreas), ORR was 14% (4/29, 95% CI: 3.9–31.7%); three responders had pathogenic germline alterations in *BRCA1/2* or *PALB2*, and the 4th had a pathogenic *TP53* mutation and *BRCA2* VUS. Of 11 patients who had a response of stable disease (SD), 4 were durable (≥6 months), of whom three had germline or somatic *BRCA2* aberrations (including one VUS, Fig. 2). The overall DCR was 20% (8/40, 95% CI: 9.1–35.7%). Two objective responses were observed in patients who had received prior platinum (2/32) (Supplementary Table 2). At the time of analysis, all responses had ended, and all patients had discontinued study treatment.

### Sequencing of post-progression biopsies reveals on-target reversion mutations

Two patients in the study consented to re-biopsy on disease progression after initial evidence of clinical response or benefit, enabling the comparison of pre-study and post-relapse tumor genotypes from targeted exome sequencing.

**Fig. 4:**
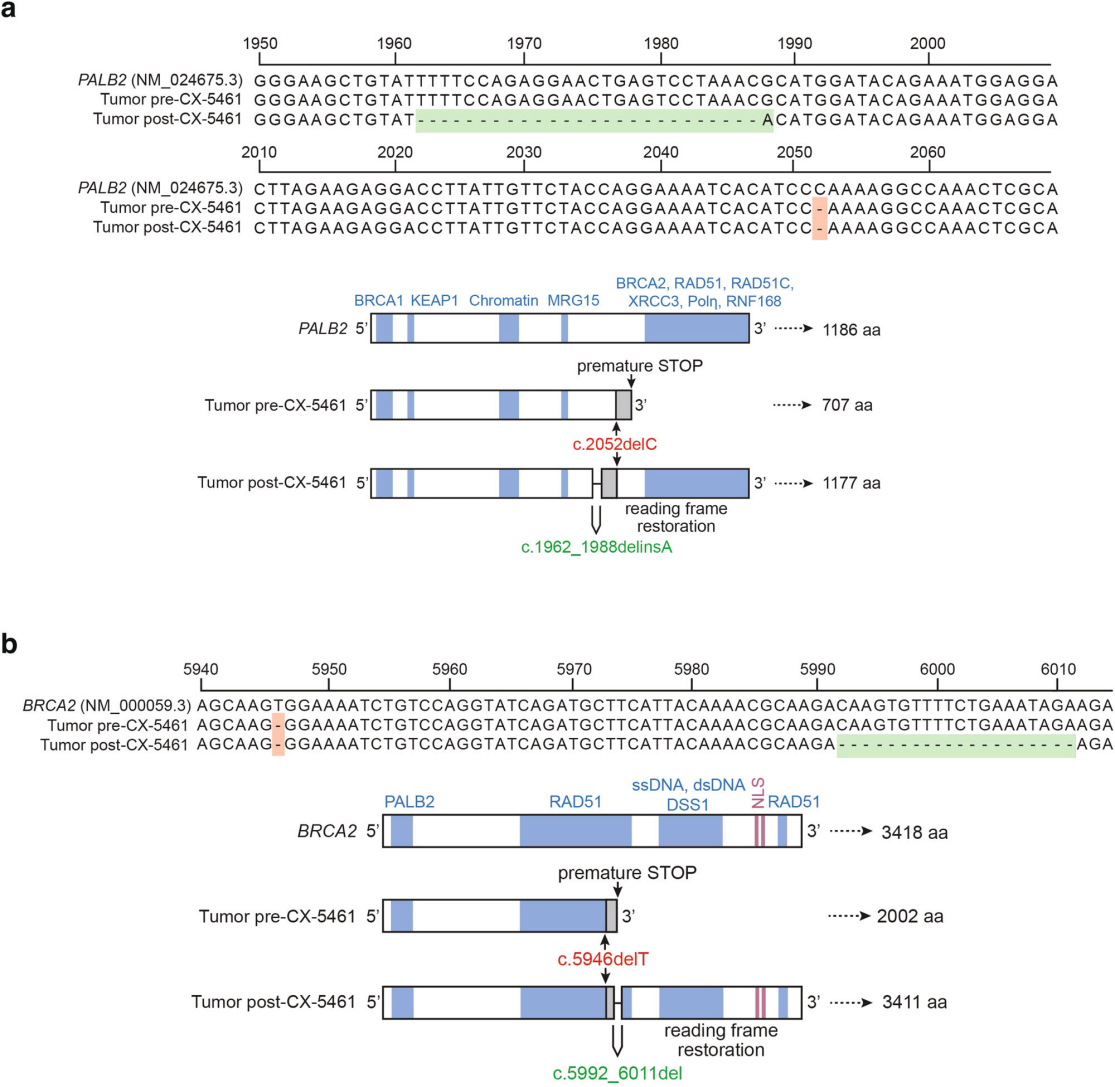
Post-progression reversion mutations. **a** *gPALB2* patient. Sequence alignment from the *PALB2* locus comparing pre- (top) and post-treatment (bottom) sequence in a responding patient (confirmed PR) with *gPALB2*-related breast cancer. Germline alteration is shown in red, and upstream acquired somatic deletion is shown in green (upper panel). The secondary mutation is predicted to restore a near full-length PALB2 protein (lower panel). **b** *gBRCA2* patient. Sequence alignment from the *BRCA2* locus comparing pre- (top) and post-treatment (bottom) sequence in a patient with *gBRCA2*-related pancreatic cancer, who experienced clinical benefit (best response SD; 13% shrinkage in target lesions, with improvement in tumor markers and disease-related symptoms), followed by progression. Germline alteration is shown in red, and downstream acquired somatic deletion is shown in green (upper panel). The secondary mutation is predicted to restore a near full-length BRCA2 protein (lower panel).

One participant with metastatic breast cancer and confirmed PR had a known pathogenic germline *PALB2* frameshift mutation in exon 5, *PALB2* c.2052delC (p.Arg686fs) diagnosed by clinical targeted panel sequencing (Figs. 2, 4a). At the time of progression, a biopsy of the growing target liver lesion revealed an additional exon 5 deletion/insertion *PALB2* c.1962_1988delinsA (p.F655Hfs*51), which restores the reading frame of *PALB2*. The appearance of a restoring frameshift is a typical resistance mechanism for HRD pathway synthetic lethal drugs^14^.

A second participant with metastatic pancreas cancer and known pathogenic *BRCA2* germline mutation (c.5946delT;p.S1982fs; rs80359550) had the best response of SD with evidence of clinical benefit (reduction in tumor markers and improvement in symptoms) (Figs. 2, 4b). At the time of disease progression, a peritoneal biopsy was obtained and subjected to targeted NGS, which revealed an acquired somatic reversion mutation, predicted to restore the BRCA2 protein reading frame (c.5992_6011delCAAGTGTTTTCTGAAATAGA p.Q1998Rfs*2).

The detection of acquired mutations predicted to restore HR function arising with the development of CX-5461 resistance in these patients provides strong evidence to support HRD as the mechanism underlying initial drug sensitivity.

### UV photosensitization is independent of G4 binding activity

**Fig. 5:**
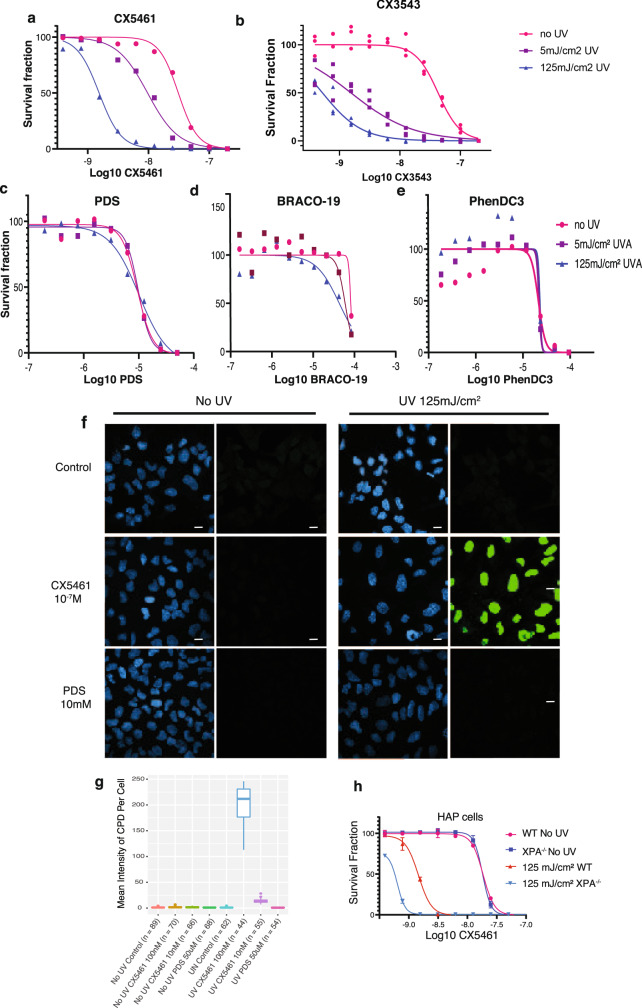
Comparison of G4 ligand photosensitization. HCT116 cells cytotoxicity (WST-1 assay) with (squares 5 mJ/cm^2^, triangles 125 mJ/cm^2^) and without (circles) UVA radiation after exposure to different G4 ligands CX-5461 (**a**), CX-3543 (**b**), PDS (**c**), BRACO-19 (**d**), and PhenDC3 (**e**). **a**–**e** Representative experiments from triplicate biological repeats are displayed as individual data points with fitted sigmoid curves. Vertical axis, fractional survival, horizontal axis log10 drug concentration (M) **(f)** Representative images of CPD immunofluorescent staining under UV and drug treatment conditions for U2OS cells, quantified from biological duplicates in (**g**) (**h**) Scale bar = 100um. U2OS cells were treated with drug or vehicle control, then immediately irradiated with UVA 125 mJ/cm^2^; and fixed 1 h later for immunofluorescence staining with CPD antibody. **g** Distribution of mean intensity of CPD level per cell (total n = in labels), with two biological repeats per condition. Vertical axis, mean CPD intensity per cell, horizontal axis, conditions (**h**) WT or KO *XPA* HAP1 cells assayed for cytotoxicity. Vertical axis, fractional survival (WST-1 assay). The horizontal axis, log10 drug concentration (M). Error bars = standard deviation of the mean. Three biological repeats per condition.

Since UV sensitization was the dominant clinical adverse effect of CX-5461, we investigated whether this is observed with all G4 ligands or is specific to CX-5461 and its chemotypes. In these experiments, we used non-HR-deficient models, since the tissues subject to clinical photosensitivity are not HR-deficient. Notably, fluoroquinolones, a class related to CX-5461, are associated with UV sensitization^15^. We evaluated the effects of CX-5461, its precursor CX-3543 (quarfloxin), and four structurally unrelated G4 binding small molecules, PDS, BRACO-19, TMPYP4, and PhenDC3 on HCT116 cell viability (Fig. 5a–e), alone or in combination with UV irradiation at 5 mJ/cm^2^ and 125 mJ/cm^2^. Strong potentiation of UVA-induced cytotoxicity, even at low UVA doses, was observed with CX-5461 and CX-3543 but not with the unrelated G4 binding compounds (Fig. 5a–e). We similarly assessed the formation of cyclobutane pyrimidine dimers (CPD), the main UVA by-product, after 1 h compound exposure and again observed strong potentiation with CX-5461 but not PDS (Fig. 5f, g). We note that DNA damage from PDS and CX-5461 is only visible after the longer time frames 4 h or more as previously described. We tested whether CX-5461 potentiates UVA-induced cell death in HAP1 cells with a knockout of *XPA*, a key mediator of CPD damage repair. In the absence of UVA radiation, WT and *XPA* deficient HAP1 cells exhibited similar sensitivity to CX-5461 (Fig. 5h). UVA radiation dramatically increased the cytotoxicity of CX-5461 in both *XPA* WT and knockout cells, to a greater extent in *XPA* knockout cells (Fig. 5h). Since DNA damage and reactive oxygen species (ROS) production are expected mediators of cell death following UVA damage, we also confirmed the pattern of CX-5461 UVA potentiation. As expected, CX-5461, but not PDS or other unrelated G4 ligands, markedly increased DNA damage (Supplemental Figure 1a, b) and ROS (Supplemental Figure 1c) in the presence of UVA. Taken together, these data show that potentiation of UV-mediated DNA damage is not a general feature of G4 ligands, but rather appears restricted to CX-5461 and its precursor.

## Discussion

CX-5461, initially developed as an RNA polymerase I inhibitor, was recently tested in a clinical trial in hematologic malignancies, where it was generally tolerated (with phototoxicity as the principal toxicity) but exhibited modest antitumor activity when administered to unselected patients at a maximum dose of 175 mg/m^2^ every 3 weeks^13^. We have recently shown through biophysical and genetic studies that CX-5461 is a G4 binder and stabilizer that induces DNA damage and is synthetic lethal with loss of key DNA-repair mechanisms, including BRCA1/2-mediated HR^3^. Furthermore, RNA-pol1 activity is not required for synthetic lethality in HRD, as structurally unrelated RNA-pol1 inhibitors exhibit no lethality in HRD backgrounds. These data and preclinical efficacy studies provided a strong rationale to evaluate CX-5461 as a G4 stabilizer in HR-deficient solid tumors. Non-clinical data, the potentially avoidable phototoxicity, and the lack of other serious toxicity observed in the first-in-human study supported the evaluation of more intensive dosing schedules. Given potential differences in safety or tolerability in this patient population, we set out to identify an RP2D using an intensified treatment schedule and to test the hypothesis that this agent would exhibit clinical activity in HRD-deficient cancers.

In our study, we were able to significantly escalate the dose of CX-5461 beyond that achieved in the first-in-human study in patients with hematologic cancers. The RP2D of 475 mg/m^2^ on days 1, 8, and 15 of a 4-week cycle, was based on phototoxicity, which occurred despite strict UV light avoidance protocols and in the absence of other significant dose-related toxicities. It should be noted that phototoxicity, including severe events, was observed at all dose levels tested, despite increasingly stringent UV avoidance recommendations. Ocular phototoxicity was observed in one patient at DL1 (100 mg/m^2^) and then at doses 475 mg/m^2^ and above and was considered dose-limiting at the highest dose level tested. Overall, phototoxicity was an important toxicity for patients, and although it resulted in only one patient discontinuing therapy, it was responsible for dose omission in six and dose reductions in two participants. Notably, 13 patients did receive radiotherapy during or following treatment with CX-5461, and no excess toxicity to ionizing radiation was noted.

Enrollment slowed during the course of the trial, mainly due to concerns regarding phototoxicity and with alternative treatments for this population, such as PARP inhibitors, becoming clinically available in Canada, which limited the addition of trial sites. The study closed before the planned expansion was completed.

Importantly, our co-clinical studies carried out to characterize phototoxicity demonstrated UVA sensitization by CX-5461—but not by other G4-stabilizing tool compounds—in vitro. These findings suggest that the photosensitization observed is not related to the mechanism-of-action, but is attributable rather to the CX-5461 chemotype. Fluoroquinolones, to which CX-5461 and its precursor CX-3543 are related, are known UV sensitizers. Coupled with the absence of other significant toxicities observed in this trial, the results of our clinical and non-clinical work raise the possibility that alternative G4-stabilizers could exhibit a broad therapeutic index.

Consistent with the results we previously reported in preclinical models^3^, this trial demonstrates that CX-5461 is active in patients with HR-deficient cancers. Four partial responses were identified, including three in patients with breast cancer— all of whom had germline DNA-repair abnormalities (2 *BRCA2*, 1 *PALB2*, 1 *TP53*). While the small number of responders limits the available analyses, the detection of reversion mutations predicted to restore HR capacity at the time of disease progression, in patients with both germline *PALB2* and *BRCA2* mutations, is strong evidence for the synthetic lethal mechanism underlying this therapeutic strategy^14^. Not all patients in our study with an identified *BRCA* mutation responded to therapy. In some cases, this is likely related to the absence of a true homologous recombination deficiency phenotype (e.g., *BRCA1* mutation in small bowel adenocarcinoma)^16^. In other cases, prior therapy with platinum or PARP inhibitors may have conferred cross-resistance with CX-5461 via restoration of functional HR or alternative mechanisms^17^. Our study was not designed or powered to fully address treatment sequencing with these agents, or to precisely define the biomarker approach. Future clinical studies should address these questions and could consider expansion of selection biomarkers, based on additional synthetic lethal partners recently described^18^.

Finally, our clinical results provide important context in light of recent preclinical reports^19,20^ that CX-5461 (and other G4 ligands such as PDS) induces topoisomerase 2 (Topo2) trapping as part of the mechanism leading to double-stranded breaks and cytotoxicity^21,22^. In this study, CX-5461 had clinical activity without evidence of the characteristic toxicities (e.g., myelosuppression and alopecia) of topoisomerase inhibitors. Furthermore, the response associations and HRD gene reversions seen here have not been reported clinically for topoisomerase inhibitors. Thus, both the antitumor activity and toxicity profiles support the notion that CX-5461 acts through a mechanism distinct from existing topoisomerase therapies. Future mechanistic studies should address whether and how G4 stabilization induces Topo2 trapping. Given our data suggesting that the dose-limiting UV sensitization is secondary to the chemical structure of CX-5461, our results could inform the development of future G4 therapies. The absence of other toxicities and available predictive biomarker strategy would enable rational development of both monotherapy and combination strategies with such agents.

## Methods

### Study oversight

This study was performed in accordance with the Declaration of Helsinki and the principles of Good Clinical Practice. The protocol was approved by Health Canada and the research ethics board for each participating center, as well as the Ontario Cancer Research Ethics Board and UBC BC Cancer Research Ethics Board. Additional correlative studies were approved by the UHN research ethics board (14-8358). All patients provided written informed consent before any study procedures were performed. The study was designed, conducted, and sponsored by the Canadian Cancer Trials Group (CCTG). Patients were accrued between 13 June 2016 and 26 August 2019.

### Study population

Patients provided written informed consent and were enrolled at three CCTG centers in Canada. Eligible patients were ≥18 years old, had an Eastern Cooperative Group (ECOG) performance status (PS) of 0–2, and had incurable solid malignancies; there was no limit on prior systemic therapy, including PARP inhibitors for the dose-escalation phase, or platinum-based chemotherapy. We planned to enroll patients with breast cancer, known *BRCA1/2* germline aberrations, or selected other HRD aberrations (e.g., *PALB2*), 1–3 prior cytotoxic regimens for advanced disease, and RECIST 1.1 measurable disease^23^ into an expansion phase at the RP2D.

Patients with asymptomatic brain/spinal cord metastasis not requiring therapy were eligible. Patients with known photosensitivity, active infections, untreated/uncontrolled cardiovascular conditions, and a history of other malignancies (except adequately treated non-melanoma skin cancer or in-situ cancer) within 2 years were not eligible. Full eligibility criteria can be found on clinicaltrials.gov (NCT02719977).

### Study objectives

The study was originally designed to identify an RP2D using a day 1 and 8 schedule and then evaluate antitumor activity in patients with probable HRD. As the dose-escalation phase was extended to evaluate much higher than expected dose levels, the protocol was amended to focus on defining an optimal biologic dose, with a small expansion at the RP2D. Secondary endpoints were to establish the safety, tolerability, and pharmacokinetics of CX-5461. Exploratory objectives included the evaluation of HRD aberrations (germline and tumor), including ctDNA and skin biopsies as predictive biomarkers of efficacy and toxicity. Companion laboratory studies were conducted to evaluate the mechanisms underlying the clinical observations.

### Study design

Doses were escalated using a 3 + 3 design, which allowed 3 or 4 patients to be initially enrolled in each dose level. CX-5461 was administered as a 60-minute intravenous infusion on day 1 (d1) and 8 q4w in dose levels 0-6 and d1, 8, and 15 q4w for dose levels 7-9. The MAD was defined as the dose level at which ≥2/3 or ≥2/6 patients experienced dose-limiting toxicity. The RP2D was defined as the next lower dose below the MAD.

DLT included the following drug-related AEs occurring during cycle 1: grade 3 phototoxicity or grade 2 phototoxicity with blistering lasting ≥7 days, if adequate prevention was used; other grades 3 or 4 non-hematologic toxicity (excluding inadequately managed nausea and vomiting, alopecia or grade 3 fatigue lasting <7 days); grade 4 myelosuppression ≥7 days, febrile neutropenia or ≥grade 3 thrombocytopenic bleeding; or other toxicities of concern including those requiring ≥14 days delay in next cycle.

The starting dose was 50 mg/m^2^ d1 and 8. An expansion cohort at the RP2D of 10-20 patients with breast cancer and *BRCA1/2* (germline) or relevant somatic aberrations was planned.

### Safety assessments

AEs were assessed according to National Cancer Institute Common Terminology Criteria for Adverse Events (CTCAE) v4.0.

### Statistical considerations

For the RP2D expansion, CX-5461 would be considered promising if two or more objective responses were observed from 10 patients. The true type I error of this design is 0.09 and the power is 76%. 95% exact confidence intervals for proportion were calculated based on the method of Clopper and Pearson.

### Pharmacokinetic and correlative studies

PK was assessed on all patients in the escalation phase. Venous blood samples were collected on treatment days 1, 8, and 15 (where applicable), and plasma CX-5461 was analyzed with HPLC-MS/MS (schedule details in Supplementary Table 3). PK parameters were calculated with non-compartmental methods. All patients had an available tissue block and provided consent for release. The protocol was amended to allow submission of a ctDNA sample and in a further amendment, a mandatory skin biopsy (pre-treatment and cycle 1 day 15) to study phototoxic effects. Fresh tumor biopsies were optional.

### Whole-genome sequencing

#### Sample processing and sequencing

FFPE tissue (either as 1.0 mm cores or pathologist-identified tumor or morphologically-normal regions of macro-dissected tissue sections) was first prepared using deparaffinization solution (Qiagen) as per the manufacturer’s protocol. DNA was extracted using the QIAamp® DNA FFPE Tissue kit (Qiagen) according to the manufacturer’s protocol. For fresh frozen (matched patient normal) skin samples, DNA was extracted using the DNeasy® Blood & Tissue Kit (Qiagen) according to the manufacturer’s protocol.

The library construction protocol used for FFPE genomic DNA sequencing is described in detail^24^. Briefly, after mechanically shearing the DNA, FFPE lesions were repaired and simultaneously end-repaired using the NEBNext FFPE End Repair reagent (New England Biolabs, NEB). Following A-tailing and adapter-ligating using the NEB Paired-End Sample Prep Premix Kit–A Tail and the NEB Paired-End Sample Prep Premix Kit–Ligation, respectively, the ligated products were PCR-enriched and indexed. Amplified libraries were purified and sequenced using an Illumina HiSeqX machine generating paired-end 150 bp reads. Sequenced libraries were aligned to the Hg19 (GRCh37) reference genome assembly using the BWA-mem version 0.7.6a aligner^25^. Aligned reads had a mean read depth of 43.33 (range 19.10 to 102.99).

#### Variant calling and interpretation

Strelka version 2.9.10^26^ and MutationSeq version 4.3.7^27^ were used with default parameters for the identification of single nucleotide variants (SNVs) and indels. SNVs were identified from the intersection of high-confidence SNVs called by MutationSeq (PR ≥ 0.85) and SNVs called by Strelka. SNV position was used to annotate variants with information from GENCODE version 19^28^ while position and sequence were used to annotate with variant information from the Clinvar 20200206_data_release^29^, and COSMIC version 91^30^. The effects of SNVs called by MutationSeq were predicted using SnpEff version 4.3^31^. Pipeline workflow with python 3.6.12, snakemake 3.13. For statistical analysis R3.6.1 and GraphPad-PRISM v8 were used.

SNVs and indels of interest were identified as those matching variants with Clinvar Clinical Significance terms including the words “pathogenic”, “likely pathogenic”, or “pathogenic association”. Somatic SNVs annotated with COSMIC records having FATHMM scores >0.5 were also identified as variants of interest. Additional SNVs of interest were identified as those absent from reference datasets but determined to have high potential impacts based on SnpEff predictions.

Indels lacking Clinvar or COSMIC annotations, but annotated with the GENCODE features CDS or exon, were identified as variants of interest if they resulted in stop codon insertion or deletion, or were called with a Strelka QSS score >34 and resulted in a potential frameshift in known GENCODE protein-coding gene and GENCODE known protein-coding transcript.

### Phototoxicity assays

Human epidermal keratinocytes (HEKa) cells were sourced from Thermo Fisher Scientific, and were grown in EpiLife™ Medium with HKGS. HAP WT and *XPA* knockout cell lines (Horizon Discovery) were cultured in IMDM, 10% FCS. Human HCT116 cells, U2OS cells (ATCC) were grown in McCoy’s 5 A medium with 10% FBS and L-glutamine. All cell lines are mycoplasma free and have been authenticated by STR or SNP profiling. CX-5461 was provided by Senhwa Biosciences, Inc. PDS is from Sigma-Aldrich, BRACO-19 from Alfa Chemistry, PhENDC3 is from Sigma-Aldrich, $\gamma$-H2AX antibody from Abcam (1:1000 for IF) and CPD antibody (1:1500) from Cosmo Bio Ltd. (Catalog number: CAC-NM-DAD-001). The WST-1 assay was performed as previously described^3^, $\gamma$H2AX foci staining was performed as previously described^3^. Immunofluorescence staining of CPD was performed according to the protocol from the manufacturer (Cosmo Bio Ltd).

ROS measurement with H2DCFDA: HCT116 cells were treated with vehicle, CX-5461 0.1 μM or PDS 10 μM for 1 h, then were added with H2DCFDA for 45 min before initial fluorescence measurement. Cells were then irradiated with UVA 125 mJ/cm^2^, and immediately sent for a second fluorescent measurement. The ratio of the second and the first fluorescent measurement was calculated as the level of ROS induced by UVA. Assays were performed in the 1-hour time frame within which UV damage manifests, in contrast to G4 ligand-induced damage which takes 3–4 h to be measurable.

### Efficacy assessment

Computed tomography of chest, abdomen, and pelvis was performed at baseline and every 8 weeks and as clinically indicated. All patients who had at least one post-baseline scan were included in efficacy analyses; patients with the non-target disease only were assessed for non-CR/non-PD and PD. Patients were evaluated for CR, PR, SD, or PD as defined by RECIST 1.1^23^. The objective response rate (ORR = CR+PR) and disease control rate (DCR = CR + PR + SD ≥ 6 months) are reported. Duration of response was defined as the time from when CR or PR was first documented until the first date that progressive disease was objectively documented or the time of the last disease assessment.

### Reporting summary

Further information on research design is available in the Nature Research Reporting Summary linked to this article.

## Supplementary information

Supplementary Information

Reporting Summary

## Data Availability

CCTG has a robust and compliant data sharing policy the details of which are available at https://www.ctg.queensu.ca/docs/public/policies/DataSharingandAccessPolicy.pdf. The data request form is available at https://www.ctg.queensu.ca/public/policies. Correlative Data—Genome WGS sequencing: VCF files with identified sequence variants are available via Zenodo at 10.5281/zenodo.6403006. BAM files corresponding to the sequencing are available at the European Genotype Archive (EGA) under accession #EGAS00001006173. Data are available under restricted access, the policy is described at: https://www.ctg.queensu.ca/public/policies, access can be obtained by contacting CCTG as described above for clinical data. Source data are provided in this paper.

## Source data

Source Data

## References

[1] Bryan TM, Baumann P (2011). G-Quadruplexes: from guanine gels to chemotherapeutics [Internet]. Mol. Biotechnol..

[2] Ohnmacht SA (2014). Discovery of new G-quadruplex binding chemotypes. Chem. Commun..

[3] Xu H (2017). CX-5461 is a DNA G-quadruplex stabilizer with selective lethality in BRCA1/2 deficient tumours. Nat. Commun..

[4] Tarsounas M, Tijsterman M (2013). Genomes and G-quadruplexes: for better or for worse. J. Mol. Biol..

[5] Lopes J (2011). G-quadruplex-induced instability during leading-strand replication: G-quadruplex-induced instability. EMBO J..

[6] Neidle S (2016). Quadruplex nucleic acids as novel therapeutic targets. J. Med Chem..

[7] McLuckie KIE (2013). G-quadruplex DNA as a molecular target for induced synthetic lethality in cancer cells. J. Am. Chem. Soc..

[8] Chartron E, Theillet C, Guiu S, Jacot W (2019). Targeting homologous repair deficiency in breast and ovarian cancers: biological pathways, preclinical and clinical data. Crit. Rev. Oncol. Hematol..

[9] Domchek SM (2015). Evolution of genetic testing for inherited susceptibility to breast cancer. J. Clin. Oncol..

[10] Yap TA, Plummer R, Azad NS, Helleday T (2019). The DNA damaging revolution: PARP inhibitors and beyond. Am. Soc. Clin. Oncol. Educ. Book.

[11] Tutt A (2018). Carboplatin in BRCA1/2-mutated and triple-negative breast cancer BRCAness subgroups: the TNT Trial. Nat. Med..

[12] Drygin D, O’Brien SE, Hannan RD, McArthur GA, Von Hoff DD (2014). Targeting the nucleolus for cancer-specific activation of p53. Drug Discov. Today.

[13] Khot A (2019). First-in-hman RNA polymerase I transcription inhibitor CX-5461 in patients with advanced hematologic cancers: results of a phase i dose-escalation study. Cancer Discov..

[14] Edwards SL (2008). Resistance to therapy caused by intragenic deletion in BRCA2. Nature.

[15] de Guidi G, Bracchitta G, Catalfo A (2011). Photosensitization reactions of fluoroquinolones and their biological consequences. Photochem. Photobiol..

[16] Jonsson P (2019). Tumour lineage shapes BRCA-mediated phenotypes [Internet]. Nature.

[17] Bouwman P, Jonkers J (2014). Molecular pathways: how can BRCA-mutated tumors become resistant to PARP inhibitors?. Clin. Cancer Res..

[18] Masud T. et al. Ubiquitin-mediated DNA damage response is synthetic lethal with G-quadruplex stabilizer CX-5461. *Sci. Rep*. **11**, 9812 (2021). 10.1038/s41598-021-88988-w PMC8105411 33963218

[19] Bossaert M (2021). Transcription-associated topoisomerase 2α (TOP2A) activity is a major effector of cytotoxicity induced by G-quadruplex ligands. Elife.

[20] Pan M (2021). The chemotherapeutic CX-5461 primarily targets TOP2B and exhibits selective activity in high-risk neuroblastoma. Nat Commun.

[21] Olivieri M (2020). A genetic map of the response to DNA damage in human cells. Cell.

[22] Bruno PM (2020). The primary mechanism of cytotoxicity of the chemotherapeutic agent CX-5461 is topoisomerase II poisoning. Proc. Natl. Acad. Sci. USA.

[23] Eisenhauer EA, Verweij J (2009). 11 New response evaluation criteria in solid tumors: RECIST GUIDELINE VERSION 1.1 [Internet]. Eur. J. Cancer Suppl..

[24] Haile S (2017). Automated high throughput nucleic acid purification from formalin-fixed paraffin-embedded tissue samples for next generation sequence analysis. PLoS ONE.

[25] Li H. Aligning sequence reads, clone sequences and assembly contigs with BWA-MEM [Internet]. *arXiv* Available from: http://arxiv.org/abs/1303.3997 (2013).

[26] Kim S (2018). Strelka2: fast and accurate calling of germline and somatic variants. Nat. Methods.

[27] Ding J (2011). Feature-based classifiers for somatic mutation detection in tumour–normal paired sequencing data. Bioinformatics.

[28] Frankish A (2019). GENCODE reference annotation for the human and mouse genomes [Internet]. Nucleic Acids Res..

[29] Landrum MJ (2018). ClinVar: improving access to variant interpretations and supporting evidence. Nucleic Acids Res.

[30] Tate JG (2019). COSMIC: the catalogue of somatic mutations in cancer [Internet]. Nucleic Acids Res..

[31] Cingolani P (2012). A program for annotating and predicting the effects of single nucleotide polymorphisms, SnpEff: SNPs in the genome of Drosophila melanogaster strain w1118; iso-2; iso-3. Fly.

