# Supplementary material for: Results of the phase I CCTG IND.231 trial of CX-5461 in patients with advanced solid tumors enriched for DNA-repair deficiencies
Source: Nat Commun. 2022 Jun 24;13:3607. doi: 10.1038/s41467-022-31199-2 (PMC9232501; doi:10.1038/s41467-022-31199-2)
Supplement: Supplementary file 1 — Supplementary Information [file 41467_2022_31199_MOESM1_ESM.pdf]

Supplementary Figures and Tables

Supplementary Figure 1. UVA radiation increased double-stranded DNA breaks and ROS.
(a) g-H2AX intensity was synergistically increased with the combination of CX-5461 and UVA radiation. Representative pictures of g-H2AX immunofluorescent staining with and without UVA radiation, for control, CX5461 10^{-8}M, CX5461 $5x10^{-8}$M, PDS 20 x 10^{-6}M , representative images from 30 fields per condition, quantified in **(b)** Quantile and mean intensity of g-H2AX level per cell, vertical axis. UV and drug factor conditions, horizontal axis. Experiments were repeated two times (biological duplicates) and similar results were obtained. Number of cells as in Figure 5g (c) The ratio of ROS level measured after UVA irradiation versus before UVA irradiation was calculated. Biological triplicates. The horizontal bar = median. ***P<0.0001, Welch's t-test; n=8

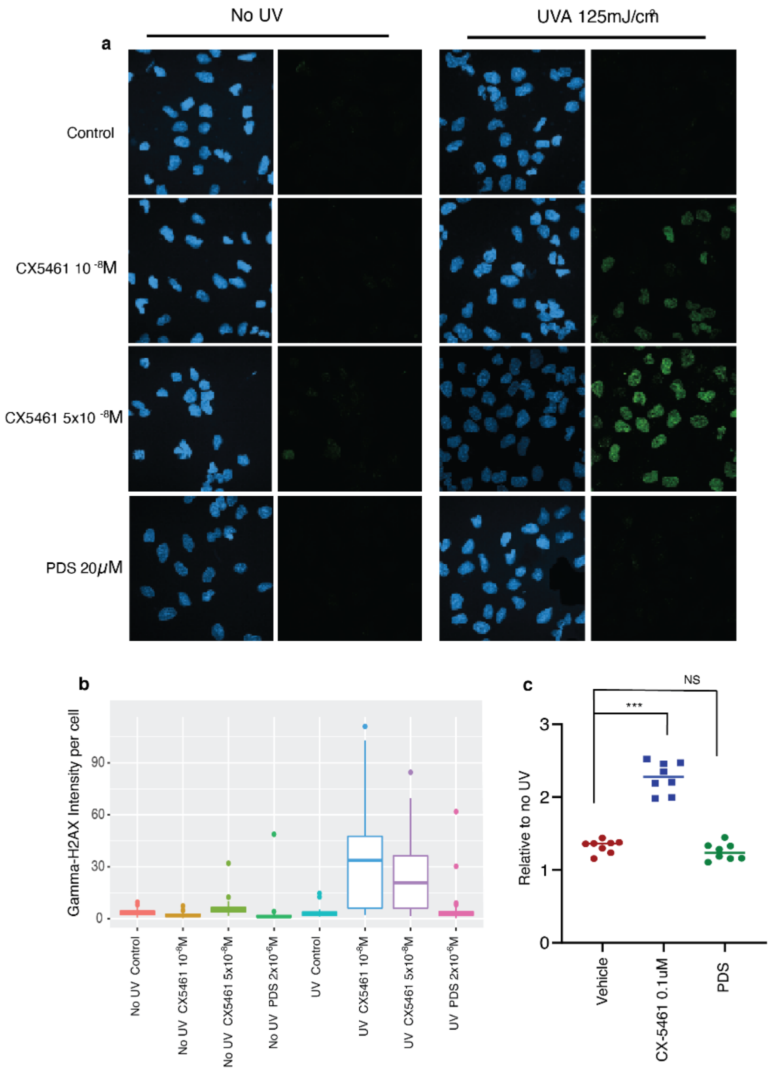

Supplementary Figure

Supplementary Table 1: Germline and somatic genotypes

PATIENT	BRCA1_CLINICAL_GERMLINE	BRCA1_CLINICAL_SOMATIC	BRCA2_CLINICAL_GERMLINE	BRCA2_CLINICAL_SOMATIC	PALB2_CLINICAL_GERMLINE	BRCA1_WGS	BRCA2_WGS	PALB2_WGS	PIK3CA_WGS	RAD51_WGS	TP53_WGS
400001	NA	NA	NA	NA	NA	NA	NA	NA	NA	NA	NA
400002	NA	NA	NA	NA	NA	NA	NA	NA	NA	NA	NA
400003	WT	NA	PROB-PATH	NA	NA	NA	NA	NA	NA	NA	NA
400004	WT	NA	PATH	NA	NA	NA	NA	NA	NA	NA	NA
400005	NA	NA	NA	NA	NA	NA	NA	NA	NA	NA	NA
400006	NA	NA	PROB-PATH	NA	NA	NA	NA	NA	NA	NA	NA
400007	NA	NA	NA	NA	NA	NA	NA	NA	NA	NA	NA
400008	PROB-PATH	NA	WT	NA	NA	NA	NA	NA	NA	NA	NA
400009	NA	PATH	NA	NA	NA	NA	NA	NA	NA	NA	NA
400010	PROB-PATH	NA	WT	NA	NA	SNV germline	reference	reference	SNV germline	reference	SNV somatic
400011	WT	NA	PATH	NA	VUS	reference	INDEL germline	SNV germline	SNV somatic	reference	SNV somatic
400012	NA	NA	PATH	NA	NA	reference	SNV unmatched_normal	reference	reference	reference	reference
400013	WT	NA	PROB-PATH	NA	NA	reference	INDEL germline	reference	reference	reference	reference
400014	WT	NA	PATH	NA	NA	reference	INDEL unmatched_normal	reference	reference	reference	reference
400015	NA	NA	NA	NA	NA	SNV germline	reference	reference	reference	reference	reference
400016	WT	NA	PATH	NA	NA	SNV germline	INDEL germline	reference	reference	reference	reference
400017	NA	NA	NA	NA	NA	SNV unmatched_tumour_sample	reference	reference	reference	reference	reference
400018	WT	NA	PATH	NA	NA	NA	NA	NA	NA	NA	NA
400019	WT	NA	PATH	NA	NA	NA	NA	NA	NA	NA	NA
400020	PATH	NA	WT	NA	NA	INDEL germline	reference	reference	reference	reference	SNV somatic
400021	WT	NA	WT	NA	NA	reference	reference	reference	reference	reference	SNV unmatched_tumour_sample
400022	NA	NA	NA	NA	NA	reference	reference	reference	SNV unmatched_tumour_sample	reference	SNV unmatched_tumour_sample
400023	WT	NA	WT	NA	NA	reference	reference	reference	SNV unmatched_tumour_sample	reference	SNV unmatched_tumour_sample
400024	NA	PATH	NA	NA	NA	SNV unmatched_tumour_sample	reference	reference	reference	reference	SNV unmatched_tumour_sample
400025	PATH	NA	NA	NA	NA	NA	NA	NA	NA	NA	NA
400026	WT	NA	PATH	NA	NA	NA	NA	NA	NA	NA	NA
400027	WT	NA	PATH	NA	NA	reference	INDEL germline	reference	reference	reference	reference
400028	WT	NA	VUS	NA	NA	reference	reference	reference	SNV somatic	reference	reference
400029	PATH	NA	WT	NA	NA	INDEL germline	reference	reference	reference	reference	SNV somatic
400030	WT	NA	WT	NA	PROB-PATH	reference	reference	INDEL germline	reference	reference	reference
400031	WT	NA	PATH	NA	NA	reference	INDEL germline	reference	reference	reference	reference
400032	WT	NA	WT	NA	NA	reference	reference	reference	reference	reference	reference
400033	WT	NA	WT	NA	NA	reference	reference	reference	reference	reference	reference
400034	WT	PATH	WT	PATH	NA	NA	NA	NA	NA	NA	NA
400035	WT	NA	VUS	NA	NA	reference	reference	reference	reference	reference	SNV somatic
400036	WT	NA	WT	NA	NA	NA	NA	NA	NA	NA	NA
400037	WT	NA	WT	NA	NA	NA	NA	NA	NA	NA	NA
400038	WT	NA	WT	NA	NA	NA	NA	NA	NA	NA	NA
400039	PATH	NA	WT	NA	NA	SNV germline	reference	reference	reference	SNV somatic	reference
400040	NA	NA	NA	NA	NA	NA	NA	NA	NA	NA	NA

Supplementary Table 2: Supplemental response information. 36 patients were fully evaluable for response (1 patient was not treated, and 4 patients were not reassessed)

	All treated patients N=40		BRCA1 / 2 or PALB2 germline or somatic mutations		BRCA1 / 2, PALB2 pr TP53 germline or somatic mutations		All treated patients N=40	
**Best Response**	**N patients**	**Response Duration Mo. Median (range)**	**N patients**	**Response Duration Mo. Median (range)**	**N patients**	**Response Duration Mo. Median (range)**	**Prior Platinum N (%) N= 32**	**Prior PARP Inhibitor N (%) N=8**
Complete Response (CR)	0							
Partial Response (PR)	4	6.5 (5.5-8.2)	3	6.3 (5.5-6.8)	4	6.5 (5.5-8.2)	2	
Stable Disease (SD)	11	3.6 (2.3-10.9)	7	3.5 (3-9.2)	7	3.5 (3-9.2)	8	1
Progressive Disease (PD)	21		12		15		18	6
Inevaluable	4		3		3		4	1

Supplementary Table 3. Schedule for collection of CX5461 pharmacokinetics

Cycle and Day	Time
C1D1	Pre-infusion
	During infusion at 30 minutes
	Immediately post infusion then:
	30 minutes post infusion
	1 hour post infusion
	2 hours post infusion
	4 hours post infusion
	6 hours post infusion*
C1D2	24 hours post infusion
C1D3	48 hours post infusion
C1D4	72 hours post infusion
C1D8	Pre-infusion
C1D15	Pre-infusion
	During infusion at 30 minutes
	Immediately post infusion then
	30 minutes post infusion
	1 hour post infusion
	2 hours post infusion
	4 hours post infusion
	6 hours post infusion*
C1D16	24 hours post infusion
C1D17-19	single sample: 48-96 hours after C1D15 infusion
C2D1	Pre-infusion
* May be 6-8 hours.	

In addition to the specific time points listed above, additional samples were to be collected from consenting patients with unusual or severe toxicity, or initial PK results suggest unusual PK or exposure.
