# Supplementary material for: Results of the phase I CCTG IND.231 trial of CX-5461 in patients with advanced solid tumors enriched for DNA-repair deficiencies
Source: Nat Commun. 2022 Jun 24;13:3607. doi: 10.1038/s41467-022-31199-2 (PMC9232501; doi:10.1038/s41467-022-31199-2)
Supplement: Supplementary file 2 — Reporting Summary [file 41467_2022_31199_MOESM2_ESM.pdf]

nature portfolio

Corresponding author(s): L. Seymour

Last updated by author(s): May 31, 2022

Reporting Summary

Nature Portfolio wishes to improve the reproducibility of the work that we publish. This form provides structure for consistency and transparency in reporting. For further information on Nature Portfolio policies, see our Editorial Policies and the Editorial Policy Checklist.

Statistics

For all statistical analyses, confirm that the following items are present in the figure legend, table legend, main text, or Methods section.

n/a	Confirmed	
☐	☒	The exact sample size (*n*) for each experimental group/condition, given as a discrete number and unit of measurement
☐	☒	A statement on whether measurements were taken from distinct samples or whether the same sample was measured repeatedly
☐	☒	The statistical test(s) used AND whether they are one- or two-sided<br>*Only common tests should be described solely by name; describe more complex techniques in the Methods section.*
☐	☒	A description of all covariates tested
☐	☒	A description of any assumptions or corrections, such as tests of normality and adjustment for multiple comparisons
☐	☒	A full description of the statistical parameters including central tendency (e.g. means) or other basic estimates (e.g. regression coefficient) AND variation (e.g. standard deviation) or associated estimates of uncertainty (e.g. confidence intervals)
☐	☒	For null hypothesis testing, the test statistic (e.g. *F*, *t*, *r*) with confidence intervals, effect sizes, degrees of freedom and *P* value noted<br>*Give P values as exact values whenever suitable.*
☐	☒	For Bayesian analysis, information on the choice of priors and Markov chain Monte Carlo settings
☒	☐	For hierarchical and complex designs, identification of the appropriate level for tests and full reporting of outcomes
☐	☒	Estimates of effect sizes (e.g. Cohen's *d*, Pearson's *r*), indicating how they were calculated

Our web collection on statistics for biologists contains articles on many of the points above.

Software and code

Policy information about availability of computer code

Data collection	Clinical Data - Medidata Rave 5.6.4.157 / Correlative Data - Genomic WGS DNA sequencing: Sequenced libraries were aligned to the Hg19 (GRCh37) reference genome assembly using the BWA-mem version 0.7.6a aligner
Data analysis	Clinical Data - SAS 9.4, R 3.6.1/ Correlative Data - GraphPad v.8; Genomic DNA sequencing: Data analysis was conducted using Python 3.6.12, R 3.6.1, and Snakemake 3.13. Variant calls were made using Strelka1 2.9.10, MutationSeq 4.3.7 and SnpEff 4.3.Variants were annotated with data from the Clinvar 20200206_data_release and COSMIC version 91.

For manuscripts utilizing custom algorithms or software that are central to the research but not yet described in published literature, software must be made available to editors and reviewers. We strongly encourage code deposition in a community repository (e.g. GitHub). See the Nature Portfolio guidelines for submitting code & software for further information.

Data

Policy information about availability of data

All manuscripts must include a data availability statement. This statement should provide the following information, where applicable:

- Accession codes, unique identifiers, or web links for publicly available datasets
- A description of any restrictions on data availability
- For clinical datasets or third party data, please ensure that the statement adheres to our policy

Clinical Data - Clinica data collected from this study, including individual participant data and a data dictionary defining each field, will be made available to interested researchers. The Canadian Cancer Trials Group (CCTG) has an established request procedure and interested investigators should submit a brief proposal using the Request for Data Proposal Form available. CCTG has a robust and compliant data sharing policy available at https://www.ctg.queensu.ca/docs/public/policies/DataSharingandAccessPolicy.pdf.   The data request form is available at https://www.ctg.queensu.ca/docs/public/policies. Upon approval, de-identified

individual participant data and relevant study documents (protocol and case report forms) will be made available. CCTG early clinical trial protocols contain drug and other information that is subject to confidentiality contractual obligations and per our SOPs we do not publish. / Correlative Data - Genome WGS sequencing: VCF files with identified sequence variants are avalable via Zenodo at doi:10.5281/zenodo.6403006 BAM files corresponding to the sequencing are available at the European Genotype Archive (EGA) under accession# EGAS00001006173. [https://ega-archive.org/studies/EGAS00001006173]. The data is available under restricted access, the policy is described at (CCTG website), access can be obtained by contacting CCTG as described above for clinical data.

Field-specific reporting

Please select the one below that is the best fit for your research. If you are not sure, read the appropriate sections before making your selection.

[x] Life sciences [ ] Behavioural & social sciences [ ] Ecological, evolutionary & environmental sciences

For a reference copy of the document with all sections, see nature.com/documents/nr-reporting-summary-flat.pdf

Life sciences study design

All studies must disclose on these points even when the disclosure is negative.

Sample size	Standard 3+3 design was used to determine sample size for phase I dose escalation and the sample size for RP2D expansion was determined by considering CX5461 is of no interest if its true response rate is 5% or lower and promising if its true response rate is 25% or higher.
Data exclusions	The data from one patient whom was withdrawn prior to receiving treatment were excluded from all analyses.
Replication	All clinical data were verified through our standard auditing and monitoring program. All cell line experiments were from 3 independent biological replicates, except where stated as two replicates, Figure 5g
Randomization	No randomization because the study was single arm phase I study to determine RP2D.
Blinding	Blinding was not relevant since there is only one treatment group.

Reporting for specific materials, systems and methods

We require information from authors about some types of materials, experimental systems and methods used in many studies. Here, indicate whether each material, system or method listed is relevant to your study. If you are not sure if a list item applies to your research, read the appropriate section before selecting a response.

Materials & experimental systems

n/a	Involved in the study	
[ ]	[x]	Antibodies
[ ]	[x]	Eukaryotic cell lines
[x]	[ ]	Palaeontology and archaeology
[x]	[ ]	Animals and other organisms
[ ]	[x]	Human research participants
[ ]	[x]	Clinical data
[x]	[ ]	Dual use research of concern

Methods

n/a	Involved in the study	
[x]	[ ]	ChIP-seq
[x]	[ ]	Flow cytometry
[x]	[ ]	MRI-based neuroimaging

Antibodies

Antibodies used	-H2AX S-129 antibody from Abcam (ab81299) and CPD antibody from Cosmo Bio Ltd. (Catalog number: CAC-NM-DAD-001)
Validation	ab81299: recombinant rabbit monoclonal ab validated by western blot and IF.<br>CAC-NM-DAD-001: monclonal Ab produced by hybridoma, validated by IF and blotting as 1) The antibodies bind to CPDs in single-stranded DNA.2) The antibodies bind to CPDs formed in every dipyrimidine sequence (TT, TC, CT and CC). 3) The antibodies stably binds to CPDs formed in oligonucleotides consisting of more than eight bases.

Eukaryotic cell lines

Policy information about cell lines

Cell line source(s)	HCT116 cells, U2OS cells (ATCC). HAP1 WT and XPA cells (Horizon Discovery, Cambridge UK).
Authentication	authenticated by STR or SNP profiling.
Mycoplasma contamination	All cell lines are mycoplasma free by PCR assay

Commonly misidentified lines (See ICLAC register)	None were used.

Human research participants

Policy information about studies involving human research participants

Population characteristics	A summary of patient characteristics is presented in Table 1 of the manuscript.
Recruitment	CRA in participating centres approached patients who were potentially eligible for this study and patients were enrolled after they provided written informed consent and the eligibility criteria were confirmed. Participants were enrolled from the patients treated at the participating institutions, or referred for participation in a clinical trial. While participants were enrolled in public cancer centres in a universally-funded healthcare system, those referred may not be representative of the broader patient population with metastatic breast cancer.
Ethics oversight	Protocol was approved by REB of participating centres, the Ontario Cancer Research Ethics Board and UBC BC Cancer Research Ethics Board). Patients provided written informed consent.

Note that full information on the approval of the study protocol must also be provided in the manuscript.

Clinical data

Policy information about clinical studies

All manuscripts should comply with the ICMJEguidelines for publication of clinical research and a completedCONSORT checklist must be included with all submissions.

Clinical trial registration	NCT02719977
Study protocol	Full protocol can be requested from CCTG Operation and Statistics Office.
Data collection	Data are collected by CRA at each participating centres. Time of enrollment period was from June 13, 2016 to November 25, 2019 and data collection period is from June 13, 2016 to January 30, 2020.
Outcomes	The primary outcome is RP2D. Safety and tolerability and pharmacokinetics of CX5461 are secondary outcomes.
